# Cytotoxic Activity of Kenaf Seed Oils from Supercritical Carbon Dioxide Fluid Extraction towards Human Colorectal Cancer (HT29) Cell Lines

**DOI:** 10.1155/2013/549705

**Published:** 2013-03-31

**Authors:** Siti Aisyah Abd Ghafar, Maznah Ismail, Latifah Saiful Yazan, Sharida Fakurazi, Norsharina Ismail, Kim Wei Chan, Paridah Md Tahir

**Affiliations:** ^1^Nutricosmeceutical and Nutrigenomic Programme, Laboratory of Molecular Biomedicine, Institute of Bioscience, Universiti Putra Malaysia, 43400 Serdang, Selangor Darul Ehsan, Malaysia; ^2^Faculty of Medicine and Health Sciences, Universiti Putra Malaysia, 43400 UPM Serdang, Selangor Darul Ehsan, Malaysia; ^3^Laboratory of Vaccines and Immunotherapeutics, Institute of Bioscience, Universiti Putra Malaysia, 43400 Serdang, Selangor Darul Ehsan, Malaysia; ^4^Institute of Tropical Forestry and Forest Products (INTROP), Universiti Putra Malaysia, 43400 UPM Serdang, Selangor, Malaysia

## Abstract

Kenaf (*Hibiscus cannabinus*) from the family Malvaceae, is a valuable fiber plant native to India and Africa and is currently planted as the fourth commercial crop in Malaysia. Kenaf seed oil contains alpha-linolenic acid, phytosterol such as **β**-sitosterol, vitamin E, and other antioxidants with chemopreventive properties. Kenaf seeds oil (KSO) was from supercritical carbon dioxide extraction fluid (SFE) at 9 different permutations of parameters based on range of pressures from 200 to 600 bars and temperature from 40 to 80°C. They were 200/40, 200/60, 200/80, 400/40, 400/60, 400/80, 600/40, 600/60, and 600/80. Extraction from 9 parameters of KSO-SFE was screened for cytotoxicity towards human colorectal cancer cell lines (HT29) and mouse embryonic fibroblast (NIH/3T3) cell lines using MTS assay. KSO-SFE at 600/40 showed the strongest cytotoxicity towards HT29 with IC_50_ of 200 *µ*g/mL. The IC_50_ for NIH/3T3 was not detected even at highest concentration employed. Cell cycle analysis showed a significant increase in the accumulation of KSO-SFE-treated cells at sub-G1 phase, indicating the induction of apoptosis by KSO-SFE. Further apoptosis induction was confirmed by Annexin V/PI and AO/PI staining.

## 1. Introduction

Kenaf (*Hibiscus cannabinus L,* family Malvaceae) is a valuable fiber plant native to India and Africa [1]. The plant that is composed of various active components including tannins, saponins, polyphenolics, alkaloids, essential oils, and steroids has long been prescribed in traditional folk medicine in Africa and India [2]. Kenaf seeds yield vegetable oil that is edible for human consumption [1]. The oil contains vitamin E with high antioxidant, *β*-sitosterol with anticancer effects, and alpha-linolenic acid (ALA), as the essential omega-3 fatty acid with anti-inflammatory and antithrombotic activity. The 3 main bioactive compounds (vitamin E, *β*-sitosterol, and ALA) in kenaf seed oil are known to contribute greatly as chemopreventive agents [3–5]. 

From the conventional method, kenaf seed oil can be extracted out using organic solvents such as n-hexane or petroleum ether. However, the oils extracted from solvent-extraction method are usually doubted for their safety due to the presence of solvent residue. Therefore, supercritical fluid extraction (SFE) seems to be the safest way to extract the oil. A supercritical fluid is any substance at a temperature and pressure above its thermodynamic critical point. It has a unique ability to diffuse through solids like a gas and dissolve materials like a liquid [6]. Additionally, it can readily change in density upon minor changes in temperature or pressure. Compared with liquid solvents, SFE has several more advantages: (1) the dissolving power of a supercritical fluid solvent depends on its density which is highly adjustable by changing the pressure or/and temperature; (2) supercritical fluid has a higher diffusion coefficient and lower viscosity and surface tension than a liquid solvent, leading to a more favorable mass transfer. These properties make it suitable as a substitute for organic solvents in a process called supercritical fluid extraction (SFE) [6]. Previous study has shown that kenaf seed oil extracted by SFE (KSO-SFE) is cytotoxic towards human cervical cancer cell line [7].

However, there has been lack of study that was conducted on oil extracted from kenaf seed using SFE technology towards colon cancer. Therefore, this study determined the cytotoxicity of KSO-SFE on colon cancer cells.

## 2. Materials and Methods

### 2.1. Materials and Chemicals

Kenaf seed was purchased from the National Kenaf and Tobacco Board (NKTB), Pasir Putih, Kelantan. Kenaf seed was cleaned and dried at constant temperature (50°C) overnight in an oven (FD 115, Fisher Scientific). The final moisture content of the dried seed was less than 5%. The dried seeds were stored at 4°C until further use. Human colorectal cancer (HT29) and mouse embryonic fibroblast (NIH/3T3) cell lines were purchased from the American Type Culture Collection (ATCC), USA. Dulbecco's modified eagle medium (DMEM), fetal bovine serum (FBS), penicillin, streptomycin, trypsin, sodium bicarbonate, propidium iodide, RNase A, Acridine orange, trypan blue, and phosphate buffer saline (PBS) were purchased from Sigma-Aldrich Co. (Sigma-Aldrich Co., St. Louis, MO, USA). Methyl thiazolyl tetrazolium (MTS) and Annexin V-FITC apoptosis detection kit were purchased from Promega (Southampton, UK). 

### 2.2. Extraction of Kenaf Seed Oil

Kenaf seed oils were prepared using supercritical carbon dioxide fluid extractor (Thar 1000 F) at 9 different extraction parameters (pressure (bars)/temperature (°C)), which were 200/40, 200/60, 200/80, 400/40, 400/60, 400/80, 600/40, 600/60, and 600/80. This was done following a method developed in our lab as described by Chan and Ismail (2009) with slight modifications [8]. In brief, 100 g of seed was ground using a Waring blender for one min and placed into a 1 L extraction vessel. The desired temperature and pressure were then set. The flow rate of carbon dioxide (CO_2_) was set at 25 g/min and regulated by an automated backpressure regulator. The extraction started after the desired temperature and pressure were obtained. The whole extraction process lasted for 2.5 h, and the yield obtained was calculated.

For Soxhlet extraction, 50 g of kenaf seeds were ground using a Waring blender for 1 min and divided equally into 2 extraction thimbles. Each thimble was then transferred into a Soxhlet extractor (Witeg, Germany). Next, 300 mL of hexane was added to each flask. After the extractions were initiated, the solvent flow rate was adjusted manually to 7 min/cycle. Extraction processes were, respectively, terminated after 20 cycles of solvent flow for rapid soxhlet extractions (SOX/S) and 100 cycles of solvent flow for conventional soxhlet extraction (SOX/L).

For conventional ultrasonic-assisted solvent extraction (SONIC), 25 g of kenaf seed was ground and homogenized with 300 mL of hexane at 13500 rpm for 3 min (Ultra-Turrax T25 basic, IKA-Werke). Subsequently, the mixture was sonicated for 90 min (Power Sonic 505, Microprocess Controlled Benchtop Ultrasonic Cleaner). After the sonication, the mixture was filtered through filter paper (Whatman number 1), and then hexane was then removed under reduced pressure (Buchi).

### 2.3. Cell Cultures

The human colonic cancer (HT29) and mouse embryonic fibroblast (NIH/3T3) cell lines from the American Type Culture Collection (ATCC), USA, were grown in Dulbecco's modified Eagle's medium (DMEM) supplemented with 10% fetal bovine serum, penicillin (100 units/mL), and streptomycin (100 *μ*g/mL). Cells were maintained in a humidified atmosphere of 5% CO_2_ at 37°C. Confluent cells were detached using 0.25% (w/v) trypsin-EDTA. Cell number and viability were determined by using haemocytometer after staining with trypan blue.

### 2.4. Cell Viability

Approximately 1 × 10^5^ cells were seeded into 96-well plate and incubated for 24 h. Kenaf seed oils were then added into the wells, and serial dilution was performed. Negative control cells were also included (untreated with KSO-SFE). The cell viability was determined by CellTiter 96 Aqueous One Solution Assay (Promega) that contains tetrazolium compound [3-(4,5-dimethylthiazol-2-yl)-5-(3-carboxymethoxyphenyl)-2-(4-sulfophenyl)-2H-tetrazolium, inner salt] (MTS). After 72 h of incubation, 20 *μ*L of MTS solution was added into the well, and the plate was incubated for 3.5 h at 37°C in 5% CO_2_ atmosphere. Viable cells colonies would produce formazan which could be measured at 490 nm with a 96-well plate ELISA reader (Opsys, USA). Cell viability was calculated with the following formula:

(1) %  Viability=Absorbance  of  sample−Absorbance  of  blankAbsorbance  of  control−Absorbance  of  blank×100%.

### 2.5. Determination of IC_50_

A curve/graph of percentage of viability cells versus concentration was plotted from three replicates of experiment. Inhibitory concentration (IC_50_), that is defined as the KSO-SFE concentration of the tested material that results in 50% of cell death, that was determined from the cell viability curve.

### 2.6. Acridine Orange(AO)/Propidium Iodide (PI) Double Staining Morphological Analysis

Approximately 5 × 10^5^ cells/mL of HT29 were seeded into each well of a 6-well plate and incubated for 24 h at 37°C in a humidified, 5% CO_2_ atmosphere. KSO-SFE was then added into the well and incubated for 72 h. After incubation, the cells were trypsinized and washed once with phosphate buffered saline (PBS). Cell suspension (10 *μ*L) was placed on a glass slide and mixed with 10 *μ*L of AO (50 *μ*g/mL) and PI (50 *μ*g/mL). The cells were viewed under a fluorescence microscope (Leica, Germany).

### 2.7. Cell Cycle Analysis by Flow Cytometer

Approximately 5 × 10^5^ cells/mL of HT29 were seeded into each well of a 6-well plate and after 24 h of incubation KSO-SFE was added. The plate was further incubated for 24 h, 48 h, and 72 h. Next, cells were detached and fixed with 5 mL ice cold 70% ethanol at −20°C for 2 h. Subsequently, the cells were washed with 5 mL of PBS, centrifuged at 3000 rpm for 10 min, and the supernatant was discarded. The pellet was resuspended with 425 *μ*L PBS, 20 *μ*L of propidium iodide (40 *μ*g/mL) and 5 *μ*L of RNase A (100 *μ*g/mL). The mixture was then incubated in the dark at 4°C for 30 min and read by a flow cytometer (CyAN_adp_, Denmark).

### 2.8. Annexin V-Propidium Iodide (AnnV-PI) Staining Apoptosis Test

Approximately 5 × 10^5^ cells/mL of HT29 were seeded into each well of a 6-well plate and after incubation for 24 h KSO-SFE was added. The cells were then further incubated for 24 h, 48 h, and 72 h. The subsequent procedures were carried out according to the instructions provided by the manufacturer of APOPTEST-FITC kit. Briefly, cells were washed with PBS, suspended in binding buffer and then added with Annexin-V FITC (AnnV) and propidium iodide (PI) and left for 10 min. The samples were then analyzed by a flow cytometer (CyAN_adp_, Denmark).

### 2.9. Statistical Analysis

Statistical analysis were performed using Statistical Package for Social Science (SPSS) version 17, and significance was accepted at *P* < 0.05. Data presented in the study were analyzed using ANOVA, values were given as mean ± SD, and means were separated using Duncan multiple range test. 

## 3. Results 

### 3.1. Cell Viability

**Figure 1:**
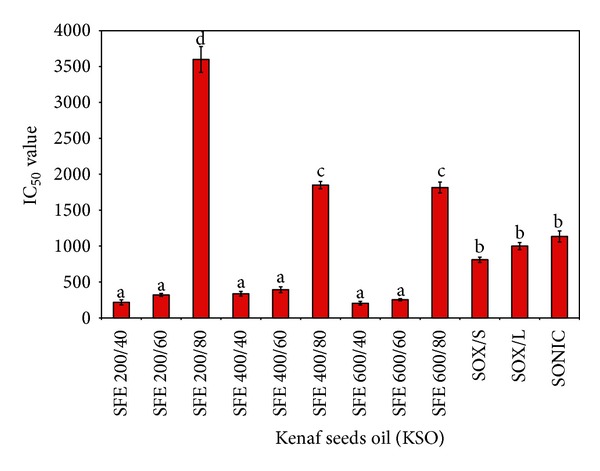
IC_50_ value of kenaf seed oil (KSO) from supercritical fluid (SFE) and solvent extraction towards HT29 cell line. The data shown are means ± SD. Means with different letter differ significantly (*P* < 0.05) using Duncan multiple range test. KSO was extracted with 9 different SFE extraction parameters (pressure (bars)/temperature (°C)). SOX/L = conventional soxhlet extraction; SOX/S = rapid soxhlet extraction; SONIC = Sonication-assisted solvent extraction.

**Figure 2:**
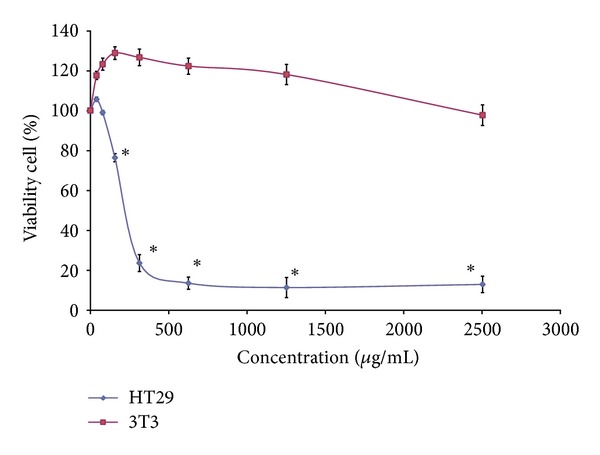
Cytotoxic effects of KSO-SFE 600/40 towards colon cancer (HT29) and normal (NIH/3T3) cells lines as detected by MTS assay after 72 h. Values are expressed as mean ± SD. An asterisk ∗ indicates  *P* < 0.05 when compared to untreated cells.

All extracts of 9 different parameters of KSO-SFE (200/40, 200/60, 200/80, 400/40, 400/60, 400/80, 600/40, 600/60, and 600/80) and solvent extracted kenaf seed oils (SOX/S, SOX/L, and SONIC) of kenaf seed were tested for cell viability through MTS assay. In general, lower IC_50_ value was observed in cells treated with KSO from SFE as compared to the one of solvent extraction (Figure 1). The lowest IC_50_ of 200 *μ*g/mL was from KSO-SFE 600/40 and was further tested on normal cell line (NIH/3T3) for cytotoxicity. Nevertheless, IC_50_ value for KSO-SFE (600/40) towards NIH/3T3 was not obtained even at the highest concentration employed (Figure 2).

### 3.2. Acridine Orange (AO)/Propidium Iodide (PI) Double Staining Morphological Analysis

**Figure 3:**
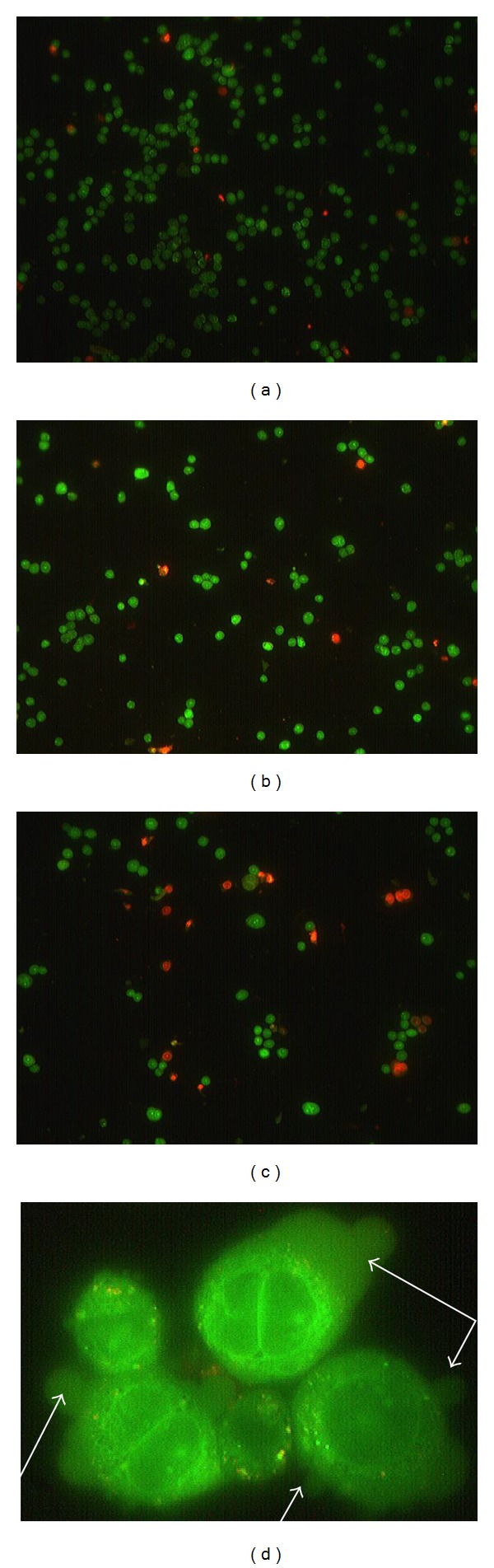
Morphological study of HT29 cell lines treated with various concentration of KSO-SFE after 72 h. (a) 0 *μ*g/mL (control); (b) 200 *μ*g/mL; (c) 1000 *μ*g/mL viewed under a fluorescence microscope (0x magnification). (d) Cell blebbing (100x magnification). Viable cells were round stained green by acridine orange, while dead cells were stained red by propidium iodide. The arrows in (d) showed blebbed cell that indicate the apoptosis characteristics. KSO-SFE: kenaf seed oil extracted by supercritical fluid extraction.

AO/PI double staining morphological analysis distinguishes viable, apoptotic, and necrotic cells. The results obtained from AO/PI double staining are shown in Figure 3. Viable cells with intact DNA and nucleus give a round and green nuclei. Nucleus of the cells undergoing apoptosis was stained green but fragmented. Late apoptotic and necrotic cells were stained orange and red. From the figure, it was clear that with the increase of KSO-SFE concentration, the number of viable cells decreased. In addition, apoptotic cells showed some other characteristics such as like plasma membrane blebbing. This indicates that most of the cell death occurred primarily through apoptosis and not necrosis.

### 3.3. Cell Cycle Analysis by Flow Cytometer

**Figure 4:**
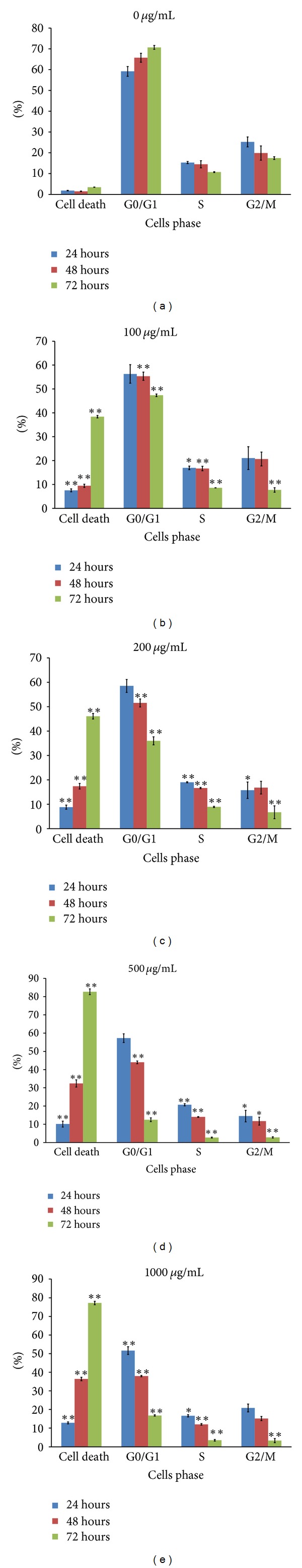
Cell cycle analysis of HT29 treated with (a) 0 *μ*g/mL; (b) 100 *μ*g/mL; (c) 200 *μ*g/mL, (d) 500 *μ*g/mL; (e) 1000 *μ*g/mL of KSO-SFE for 24 h, 48 h, and 72 h. *P < 0.05, **P < 0.01 compared to control cells.

Figure 4 shows the effects of KSO-SFE on the cell cycle of HT29 cells. A significant (*P* < 0.01) increase in the cell population at sub-G1 phase was observed at 100 *μ*g/mL of KSO-SFE (7.52%  ±  0.62) and was more pronounced at the higher concentrations (200, 500, 1000 *μ*g/mL). After 72 h, cell death was found to increase significantly up to 77.14%  ±  1.13 compared to the control (3.46%  ±  0.08) in cells treated with KSO-SFE at 1000 *μ*g/mL.

### 3.4. Annexin V-Propidium Iodide (AnnV-PI) Staining Apoptosis Test

**Figure 5:**
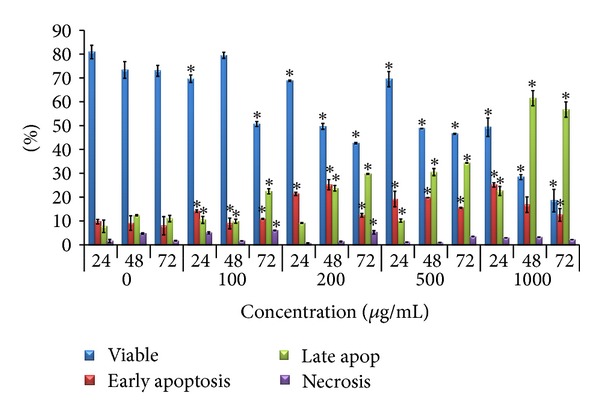
Annexin V-PI flow cytometry analysis of HT29 cells treated with 0 *μ*g/mL, 100 *μ*g/mL, 200 *μ*g/mL, 500 *μ*g/mL, and 1000 *μ*g/mL of KSO-SFE. The cells were classified into four stages: viable, early apoptosis, late apoptosis, and necrosis/secondary necrosis. Values are expressed as mean ± SD. *P < 0.05 compared to untreated cells.

Based on Figure 5, some changes in the cell population were noticed at treatment of 100 *μ*g/mL KSO-SFE and becoming more pronounced at higher concentration (200, 500, and 1000 *μ*g/mL) over time. After 24 h, only 9.73%  ±  0.92 of the untreated cells entered early apoptosis stage, but 14.15%  ±  2.17 (100 *μ*g/mL), 21.37%  ±  3.26 (200 *μ*g/mL), 19.23%  ±  0.90 (500 *μ*g/mL), and 25.15%  ±  0.66 (1000 *μ*g/mL) of treated cell have entered early apoptosis stage. After 72 h only 11.0%  ±  1.38 of untreated cells entering late apoptosis but 22.46%  ±  0.22 (100 *μ*g/mL), 29.74%  ±  0.12 (200 *μ*g/mL), 34.34%  ±  3.20 (500 *μ*g/mL), and 56.74%  ±  4.72 (1000 *μ*g/mL) of treated cells entering late apoptosis. Besides, there is no significant difference (*P* < 0.05) when compared with 1.72%  ±  0.12 of untreated cells, 3.46%  ±  0.03 and 2.18%  ±  0.16 of 500 *μ*g/mL and 1000 *μ*g/mL treated cells entering necrosis stage, respectively. The differences between the percentage of untreated cells and KSO-SFE treated cells in early apoptosis, late apoptosis, and secondary apoptosis stages were all significant (*P* < 0.05).

## 4. Discussion

There has been substantial interest in the search for potential chemopreventive agents in the past years for treatment of cancers. Understanding how dietary components regulate proliferation and cell survival could play a critical role in the development of new agents that can prevent and treat cancer with low toxicity [9]. Cancer chemoprevention was described as the use of natural synthetic chemicals allowing suppression, retardation, or inversion of carcinogenesis [10]. Apoptosis induction is one of a potent defensive strategies against cancer progression [11–14]. Numerous diet-derived agents are among promising agents and agent combinations that are being evaluated clinically as chemopreventive agents for major cancer targets including colon, breast, prostate, and lung cancers [15]. 

Yield of the oil extracted is one of the important criteria to be considered for commercialization of products such as drugs, functional foods, or dietary supplement. Apparently, a rise in the extraction pressure increased the yield of KSO from SFE according to the following sequence: 600/80 ≥ 600/60 ≥ 600/40 ≥ 400/80 ≥ 400/40 ≥ 400/60 > 200/40 > 200/60 ≥ 200/80 (*P* < 0.05). Pressure and temperature are two important SFE parameters that give huge effects to the yield of kenaf seed oil. Elevation in pressure at a given temperature results in an increase in the fluid (CO_2_) density which means that it enhanced solubility of the solutes hence, increased the yield of KSO-SFE [16]. On the other hand, at a constant pressure, the density of CO_2_ decreases when the temperature is increased. In addition, temperature also affects the volatility of the solute. Hence the effect of a temperature elevation is difficult to predict because of its dependence on the nature of the sample. For instance, a nonvolatile solute would result in lower yield, whereas for a volatile solute it will increase in yield [16].

The results of cell viability analysis showed that all 9 parameters of KSO-SFE were cytotoxic towards HT29 cells with the IC_50_ values ranging from 200 *μ*g/mL to 3750 *μ*g/mL (Figure 1). Apparently, there was a rise in the cytotoxic activity of KSO-SFE according to the following sequence: 600/40 ≥ 400/60 ≥ 400/40 ≥ 200/40 ≥ 600/60 ≥ 200/60 > 600/80 > 400/80 ≥ 200/80 (*P* < 0.05). Whereas the IC_50_ towards NIH/3T3 cells line could not be determined (Figure 2). This indicates that KSO-SFE especially the 600/40 was cytotoxic towards HT29 cell line. The pressure of 600 bars and temperature at 40°C were probably the most suitable condition where most of the bioactive components and nutritive values of kenaf seed have been successfully extracted. Kenaf seed oil was extracted at 80°C at any pressure, and the IC_50_ towards HT29 cell lines was at 3750 *μ*g/mL. The temperature might be high enough to degrade some of the bioactive components, or that some biological active substances may not be extracted at that temperature compared to others (40°C and 60°C) [17]. For soxhlet extracts [(SOX/L) and (SOX/S)], higher IC_50_ was noted when compared to the SFE extracts. The results proved the advantage using SFE than conventional methods (solvents extraction) for extraction of oil from kenaf seed. The results obtained can be used as a basis for development of a disease-oriented drug discovery. From Manosroi (2006), (1) extract that had IC_50_ value of less than 125 *μ*g/mL can be a possible candidate for further development to cancer therapeutic agent; (2) extract that had the IC_50_ value between 125 and 5000 *μ*g/mL has moderate possibility to be developed into a cancer therapeutic agent but high possibility to be developed as a chemopreventive agents and; (3) extract that had the IC_50_ more than 5000 *μ*g/mL has low possibility to be developed as a cancer therapeutic agent. Based on criteria and Figure 3(b), KSO-SFE falls into the group that has the potential to be developed as a chemopreventive agent [18]. This result has been supported by previous study showing that kenaf seed oil extracted by SFE reduced aberrant crypt foci (ACF) number on azoxymethane (AOM) induced rats [19]. For the purpose of this study, KSO-SFE of 600/40 was chosen as the most potential extract, since it has the lowest IC_50_ (200 *μ*g/mL) among others. 

It is important to determine mode of cell death induced by KSO-SFE. Since cell death could be divided into apoptosis or necrosis. Apoptosis is more favourable than necrosis because it is a programmed cell death that does not trigger inflammatory responses [13]. In AO/PI staining, DNA-binding dye acridine orange (AO) and propidium iodide (PI) were used for morphological detection of apoptotic and necrotic cells. AO intercalates into DNA giving it a green appearance. Thus, viable cells have a bright green nucleus. PI is only taken up by nonviable cells. This dye also intercalates into DNA, making it appears orange. The live cells with intact membrane have a uniform green color in their nuclei. Early apoptotic cells have chromatin condensation with bright colored nuclei. Late apoptotic cells have bright orange areas of condensed chromatin in the nucleus that distinguished them from necrotic cells, which have a uniform orange color [20, 21]. Based on the AO/PI dual staining assay, round and healthy viable cells can be seen in the control cells untreated with KSO-SFE. On the other hand, reduction in the cancer cell number and also feature of apoptosis like cell blebbing were noted in the treated cells. This provides qualitative morphological proof that KSO-SFE could induce apoptosis and inhibit cancer cell growth. This is further confirmed with the existence of the sub-G1 area by cell cycle analysis. It has been reported that appearance of sub-G1 cells is the marker of cell death caused by apoptosis [22]. 

Annexin V/PI (AnnV/PI) staining is based on ability of the protein Annexin V to bind to phosphatidylserine (PS) exposed on the outer membrane leaflet, but upon induction of apoptosis it is translocated to the outer membrane leaflet and becomes available for Annexin V binding. The addition of PI enabled viable (AnnV^−^/PI^−^), early apoptotic (AnnV^+^/PI^−^), late apoptotic (AnnV^+^/PI^+^), and necrotic (AnnV^−^/PI^+^) cells to be distinguished [23]. Based on that, it shows that KSO-SFE induced early apoptosis at 24 h and late apoptosis/secondary necrosis at 72 h necrosis in HT29 cells (Figure 5). As KSO-SFE induced cell death in sequence from early apoptosis to late apoptosis/secondary necrosis [24], it is concluded that KSO-SFE actually induces cell death via apoptotic rather than necrotic pathway.

KSO-SFE was found to contain higher vitamin E by our group compared to the one of solvent extraction, whereby when compared to solvent extraction [1, 5]. Vitamin E is known to induce apoptosis in HT29 cell line and inhibit human prostate cancer cell growth [25, 26]. KSO-SFE is also rich in sterol especially *β*-sitosterol [5]. Previous studies have shown that *β*-sitosterol reduces carcinogen-induced cancer of the colon in rats [27]. Several studies have indicated that *β*-sitosterol inhibits the growth of various cultured cancer cell lines that are associated with cell cycle arrest and the stimulation of apoptotic cell death [28–30]. Alpha-linolenic acid (ALA), the essential omega-3 fatty acid, was also found in kenaf seed oil that is known as chemopreventive agents [1, 4]. These bioactive compounds are probably responsible for the cytotoxicity of KSO-SFE in HT29 cell line. 

In conclusion, although the yield of KSO-SFE is lower than the one of solvent extraction, this study has shown that KSO-SFE possesses better cytotoxic properties towards HT29 cell line. It induced cell death via apoptosis and inhibited proliferation of the cancer cells. These findings add to a growing body of literature on health benefit of KSO-SFE. As current studies are focusing on *in vitro* KSO-SFE effects, further research might explore KSO-SFE effects *in vivo.* Finding of this study will be beneficial for further development of new chemotherapeutic/chemopreventive agents.
